# Multiple mechanisms underlying acquired resistance to taxanes in selected docetaxel-resistant MCF-7 breast cancer cells

**DOI:** 10.1186/1471-2407-14-37

**Published:** 2014-01-22

**Authors:** Harris Wang, The Vo, Ali Hajar, Sarah Li, Xinmei Chen, Amadeo M Parissenti, David N Brindley, Zhixiang Wang

**Affiliations:** 1 Department of Medical Genetics, University of Alberta, Edmonton, AB T6G 2H7, Canada; 2 Department of Biochemistry and Signal Transduction Research Group, Faculty of Medicine and Dentistry, University of Alberta, Edmonton, AB T6G 2H7, Canada; 3 Regional Cancer Program, Sudbury Regional Hospital, Sudbury, ON, Canada

**Keywords:** Breast cancer, Taxane, Doxorubicin, Chemoresistance, MCF-7 cell, ABC proteins, β-tubulin isoforms, Microtubule dynamics

## Abstract

**Background:**

Chemoresistance is a major factor involved in a poor response and reduced overall survival in patients with advanced breast cancer. Although extensive studies have been carried out to understand the mechanisms of chemoresistance, many questions remain unanswered.

**Methods:**

In this research, we used two isogenic MCF-7 breast cancer cell lines selected for resistance to doxorubicin (MCF-7_DOX_) or docetaxel (MCF-7_TXT_) and the wild type parental cell line (MCF-7_CC_) to study mechanisms underlying acquired resistance to taxanes in MCF-7_TXT_ cells. Cytotoxicity assay, immunoblotting, indirect immunofluorescence and live imaging were used to study the drug resistance, the expression levels of drug transporters and various tubulin isoforms, apoptosis, microtubule formation, and microtubule dynamics.

**Results:**

MCF-7_TXT_ cells were cross resistant to paclitaxel, but not to doxorubicin. MCF-7_DOX_ cells were not cross-resistant to taxanes. We also showed that multiple mechanisms are involved in the resistance to taxanes in MCF-7_TXT_ cells. Firstly, MCF-7_TXT_ cells express higher level of ABCB1. Secondly, the microtubule dynamics of MCF-7_TXT_ cells are weak and insensitive to the docetaxel treatment, which may partially explain why docetaxel is less effective in inducing M-phase arrest and apoptosis in MCF-7_TXT_ cells in comparison with MCF-7_CC_ cells. Moreover, MCF-7_TXT_ cells express relatively higher levels of β2- and β4-tubulin and relatively lower levels of β3-tubulin than both MCF-7_CC_ and MCF-7_DOX_ cells. The subcellular localization of various β-tubulin isoforms in MCF-7_TXT_ cells is also different from that in MCF-7_CC_ and MCF-7_DOX_ cells.

**Conclusion:**

Multiple mechanisms are involved in the resistance to taxanes in MCF-7_TXT_ cells. The high expression level of ABCB1, the specific composition and localization of β-tubulin isoforms, the weak microtubule dynamics and its insensitivity to docetaxel may all contribute to the acquired resistance of MCF-7_TXT_ cells to taxanes.

## Background

Breast cancer is the most common type of cancer in women and accounts for one third of all cancers in women. The incidence of breast cancer is continuously increasing, with more than one million reported new cases diagnosed per year worldwide [1-3]. Among these cases, 20-30% present with metastatic or locally advanced disease, and another 30% will develop recurrent or metastatic disease [3].

Chemotherapy is often used to treat breast cancer in both the adjuvant and neoadjuvant settings and often involves the administration of anthracyclines together with (or followed by) taxanes. Typical taxanes used in current treatment regimens for breast cancer include paclitaxel (Taxol) and docetaxel (Taxotere), while typical anthracyclines include doxorubicin (Adriamycin) and epirubicin (Pharmorubicin) [4,5]. Docetaxel belongs to the group of taxanes that were first introduced into clinical use during the 1990’s. Both paclitaxel and docetaxel bind to β-tubulin in assembled tubulin, thereby reducing depolymerisation [6]. Taxanes stabilise microtubules and dampens microtubule dynamics to prevent the normal formation of mitotic spindles [6]. This leads to chronic activation of the spindle assembly checkpoint (SAC), which in turn leads to mitotic arrest [7]. Extended mitotic arrest eventually leads to cell death [8]. We have recently shown that the taxanes also strongly induce TNF alpha production, which may also promote apoptosis through binding to its receptor TNFR1 [9]. On the other hand, doxorubicin is an anthracycline antibiotic, which intercalates with DNA. While the mechanisms of action are not yet completely understood, one very important component of the activity of doxorubicin is its interaction with chromatin and its constitutive components: DNA and histones. These interactions lead to chromatin unfolding and aggregation. This chromatin structural disruption is likely to interfere with DNA replication and transcription, which eventually leads to cell apoptosis. It was also suggested that doxorubicin interacts with key cellular enzymes such as topoisomerases I and II. Topoisomerase II mediated DNA damage by doxorubicin is followed by G1 and G2 growth arrest and induction of apoptosis, which correlates with tumor response and patient outcomes [1,10-12].

Resistance to chemotherapy can occur prior to drug treatment (primary or innate resistance) or develop over time following exposure to a given chemotherapeutic agent (acquired resistance) [13]. Chemoresistance presents a major obstacle to therapy and leaves few effective treatment options [5]. Both innate and acquired resistance to taxanes and doxorubicin are common as more breast cancer patients receive these drugs [1,14]. The most established *in vitro* mechanism for resistance to more than one chemically unrelated class of agents (multidrug resistance) is the overexpression of drug efflux proteins. The best known drug efflux proteins are members of the ATP-binding cassette (ABC) superfamily, including P-glycoprotein [Pgp; also called multidrug resistance protein (MDR) or ABCB1], the multidrug resistance-associated protein 1 [MRP-1, also called ABCC1], and the breast cancer resistance protein [BCRP, also called ABCG2]. ABC transporter substrates include a diverse array of compounds, many of them structurally unrelated. These proteins protect cells and tissues by exporting potential toxins, including anticancer agents from cells in normal tissues and cancer cells [4]. In general, ABCB1 transports large hydrophobic compounds, whereas ABCC1 and ABCG2 transport both hydrophobic drugs and large anionic compounds [15]. ABC proteins have been implicated in both taxane and doxorubicin resistance in breast cancers [1,3,4,14]. When 60 cell lines were tested, it was found that the lower the ABCB1 expression level, the greater the sensitivity to paclitaxel in the cell lines [16]. However, in clinic studies the results are controversial. One study shows that increased ABCB1 expression level is correlated with shortened disease-free survival [17]. Some other studies show that no correlation between ABCB expression level and response to either paclitaxel or docetaxel treatment in breast cancer patients [18]. On the other hand, both ABCC1 and ABCG2 mediate resistance to doxorubicin, but not paclitaxel [5,19].

Resistance may also arise from the expression of proteins underlying a specific drug’s mechanism of action. For example, taxanes operate by binding to β-tubulin. Taxane-resistant cancer cells may have altered expression and function of certain β-tubulin isotypes, caused by mutations in β-tubulin, and increased microtubule dynamics associated with altered microtubule-associated protein (MAP) expression [3,4,14,20-23]. Altered expression of the topoisomerase IIa gene (TOP2A), which encodes the enzyme target of the anthracyclines, may confer anthracycline resistance [24].

Chemoresistance is a major factor involved in poor response and reduced overall survival in patients with locally advanced and metastatic breast cancer. Chemoresistance is a very challenging and complex phenomenon involving a number of complex mechanisms. Elucidating these mechanisms is crucial to understanding how to improve the use of taxane and doxorubicin in cancer treatment. Although extensive studies have been carried out to understand chemoresistance in breast cancer both in vitro and clinically, many questions remain unanswered. In previous research, we established several drug-resistant MCF-7 cell lines by exposing MCF-7 cells to increasing concentrations of specific chemotherapy drugs [25]. Our study showed that while drug transporters were induced during selection for drug resistance (which reduced drug accumulation into tumour cells), additional drug-transporter-independent mechanisms must play important roles [25]. In the current study, we used two of our preciously created resistant cell lines, doxorubicin-resistant MCF-7 cells (MCF-7_DOX_) and docetaxel-resistant MCF-7 cells (MCF-7_TXT_), to study the mechanisms underlying the acquired drug resistance, with emphasis on the resistance to taxanes in MCF-7_TXT_ cells. We show that MCF-7_TXT_ cells are ten times more resistant to both docetaxel and paclitaxel than the sensitive wild type parental cell line (MCF-7_CC_). MCF-7_DOX_ cells are eight times more resistant to doxorubicin than MCF-7_CC_ cells. However, MCF-7_TXT_ cells are not cross-resistant to doxorubicin and MCF-7_DOX_ cells are not cross-resistant to taxanes. We also showed that multiple mechanisms are involved in the resistance to taxanes in MCF-7_TXT_ cells. Firstly, the selected chemo-resistant cell lines express higher levels of certain ABC proteins. The expression level of ABCB1 is very high only in the MCF-7_TXT_ cells and the expression level of ABCC1 is very high only in the MCF-7_DOX_ cells. The expression level of ABCG2 is similar in both the selected chemo-resistant and the parental MCF-7 cell lines. Moreover, MCF-7_TXT_ cells are also more resistant to taxane-induced mitotic spindle disruption and M phase arrest, which leads to apoptosis. The microtubule dynamics of MCF-7_TXT_ cells are insensitive to the docetaxel treatment, which may partially explain why docetaxel is less effective in inducing M-phase arrest and apoptosis in MCF-7_TXT_ cells in comparison with MCF-7_CC_ cells. Finally, MCF-7_TXT_ cells express relatively higher levels of β-2 and β-4 tubulin and relatively lower levels of β-3 tubulin than both MCF-7_CC_ and MCF-7_DOX_ cells. The subcellular localization of various β-tubulin isoforms in MCF-7_TXT_ cells is also different from that in MCF-7_CC_ and MCF-7_DOX_ cells.

## Methods

### Cell culture and treatment

The cell lines that were used in this study include MCF-7 breast cancer cells selected for resistance to doxorubicin (MCF-7_DOX_) and docetaxel (MCF-7_TXT_), and the non-resitant wild type parental cell line (MCF-7_CC_) as we previously described (Hembruff [25]). Detailed selection process and characterization of these selected cell lines were described previously [25]. All cells were grown at 37°C in Dulbecco's modified Eagle's medium containing 10% FBS supplemented with non-essential amino acids and were maintained in a 5% CO_2_ atmosphere. MCF-7_DOX_ cells were maintained at 95 nM doxorubicin and MCF-7_TXT_ cells were maintained at 5 nM docetaxel.

### Antibody and chemicals

Mouse monoclonal anti-ABCG2 (BXP-21), rabbit polyclonal anti-MDR (ABCB1) (H-241), and rabbit polyclonal anti-MRP1 (ABCC1) (E-19) antibodies were purchased from Santa Cruz Biotechnology, Inc. (Santa Cruz, CA). Rabbit anti-tubulin β1 and β3, and mouse monoclonal anti-tubulin β2 and β4 antibodies were purchased from Abcam (Toronto, ON). All secondary antibodies conjugated with FITC and TRITC were from Life Technologies Inc (Burlington, ON). Mammalian Protein Extraction Reagent (M-Per) was purchased from Thermo Fisher Scientific Inc. (Rockford, IL USA). Vybrant MTT Cell Proliferation Assay Kit and GFP tagged α-tubulin with the CellLight® Reagents BacMam 2.0 were from Invitrogen (Grand Island, NY). Unless otherwise specified, all chemicals were purchased from Sigma-Aldrich.

### Cytotoxicity assay

Cells were plated onto 96-well plates, 10,000 cell/well for each MCF-7 cell line. Forty-eight hours later, the culture medium was replaced by fresh medium containing various concentrations of Doxorubicin, paclitaxel and docetaxel for 24 h. The percentages of viable cells were then determined by the conversion of the water soluble MTT (3-(4,5-dimethylthiazol-2yl)-2,5-diphenyltetrazolium bromide) to an insoluble formazan, relative to drug free controls, using the Vybrant MTT Cell Proliferation Assay Kit (Invitrogen, Grand Island, NY). All cytotoxicity data shown are the means of at least three independent experiments. Similar experiment were performed with drug treatment for 72 h.

### Immunoblotting

The expression levels of various proteins were examined by immunoblotting as previously described [26]. Briefly, protein lysates from three MCF-7 cell lines treated or not treated with docetaxel were obtained by lysing cells with M-PER Mammalian Protein Extraction Reagent and a protease inhibitor cocktail. The expression of various proteins including ABCB1, ABCC1, ABCG2, β1-, β2-, β3-, and β4-tubulin was examined by immunoblotting following SDS PAGE.

### Apoptosis assay

Cell apoptosis was determined by chromatin condensation and nuclear morphology. Cells in cover slips were treated with various concentrations of drugs for 24 h. DNA was stained by DAPI (300 nM) for 5 min. Chromatin condensation and nuclear morphology were examined using a Delta Vision microscopic system. Delta Vision SoftWoRx software was used to deconvolve the images.

### Indirect immunofluorescence

Indirect immunofluorescence was carried out as previously described [26]. For the staining of ABC proteins, cells were either treated or not treated with docetaxel at indicated concentrations for 24 h. After fixation with -20°C methanol, cells were incubated with antibodies against ABCB1, ABCC1 or ABCG2, followed by FITC-conjugated secondary antibodies. Nuclei were then count stained with DAPI. For the expression and localization of α-tubulin and various β-tubulin isoforms including β1, β2, β3, and β4, cells were incubated with the antibodies against these tubulins followed by incubation with TRITC-conjugated secondary antibodies. The images were obtained using a Delta Vision microscopic system. Delta Vision SoftWoRx software was used to deconvolve the images.

### Live imaging

Live imaging was used to study docetaxel induced M-phase arrest and microtubule dynamics. As previously described [26], MCF-7_CC_ and MCF-7_TXT_ cells were cultured on 35 mm poly-L-lysine-coated coverslips (Fisher) overnight. To assay docetaxel-induced M-phase arrest, cells were incubated with DMEM containing 250 ng/ml Hoechst 33342 (Calbiochem) for 10 min to stain DNA. Then, the coverslip was mounted on a sample holder and incubated with DMEM without phenol red, supplemented with 10% FBS with or without docetaxel. To assay microtubule dynamics, the cells were transfected with GFP tagged α-tubulin with the CellLight® Reagents BacMam 2.0 (Invitrogen, Grand Island, NY) for 24 h according to the Manufacturer’s instruction. The coverslip was then mounted on a sample holder and incubated with DMEM without phenol red, supplemented with 10% FBS either with or without docetaxel of indicated concentrations for 1 h.

Experiments were performed in a chamber maintained at 37°C and 5% CO2. The fluorescence images were acquired at various time intervals for indicated time using a Delta Vision microscopic system. Delta Vision SoftWoRx software was used to deconvolve images and generate movies. To calculate the extension and shortening rate of the microtubules, we followed the microtubules that were either growing or shrinking. For each datum, at least 20 microtubules from 8 cells were measured.

## Results

### Cross-resistance of MCF-7_CC_, MCF-7_DOX_, and MCF-7_TXT_ cells to various cancer drugs

**Figure 1:**
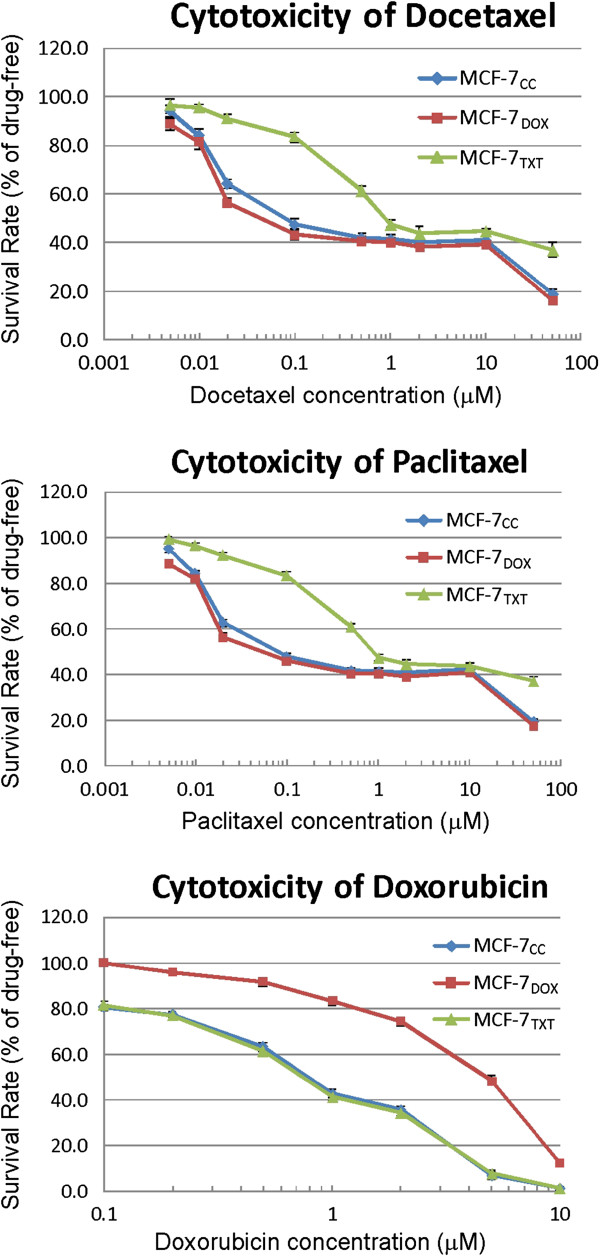
**Dose-response curve to drug treatment.** Cytotoxicity of paclitaxel, docetaxel and doxorubicin at various concentrations for MCF-7_CC_, MCF-7_TXT_ and MCF-7_DOX_ cells was determined following 24 h drug treatment as described in Methods. The means of at least three independent experiments are plotted. The error bar is the standard error; most of them are smaller than the symbols of the plot.

We first determined whether the selected drug resistant cell lines show cross-resistance to different drugs. The three cell lines used in the experiments include Docetaxel-resistant MCF-7 cells selected with increased docetaxel concentration (MCF-7_TXT_), Doxorubicin-resistant MCF-7 cells selected with increased doxorubicin concentration (MCF-7_DOX_), and the parent non-resistant MCF-7 cell line (MCF-7_CC_) [25]. The sensitivity of MCF-7_CC_, MCF-7_TXT_ and MCF-7_DOX_ cells to various drugs was determined by using a cytotoxicity assay based on the Vybrant MTT Cell Proliferation Assay following drug treatment for 24 h (Figure 1) or 72 h (data not shown). The patterns were similar for both 24 and 72 h drug treatment, but 72 h treatment shifted the curve downward. Based on the dose-response curve from this cytotoxicity assay, the IC_50_s for various drugs were calculated for the above cell lines (Table 1). As shown in Table 1, MCF-7_DOX_ cells were nearly 8 times more resistance to Doxorubicin than MCF-7_CC_ cells; however, MCF-7_DOX_ cells showed no resistance to paclitaxel and docetaxel. In fact MCF-7_DOX_ cells are slightly more sensitive to paclitaxel and docetaxel than MCF-7_CC_ cells. Similarly, MCF-7_TXT_ cells were 10 times more resistant to paclitaxel and docetaxel than MCF-7_CC_ cell, but showed no resistance to Doxorubicin. These data indicate that docetaxel-resistant MCF-7_TXT_ cells are cross-resistant to paclitaxel. However, MCF-7_TXT_ and MCF-7_DOX_ cells show no cross-resistance to doxorubicin and taxanes, respectively.

**Table 1:** **IC50 (mM) of paclitaxel, docetaxel and doxorubicin for MCF-7wt, MCF-7txt and MCF-7dox cells (calculated from Figure**1**)**

	**Docetaxel**	**Paclitaxel**	**Doxorubicin**
MCF-7wt	0.08	0.07	0.8
MCF-7txt	0.9	0.8	0.8
MCF-7dox	0.04	0.04	5

### Expression and subcellular localization of ABC proteins

The most established *in vitro* mechanism for resistance to more than one chemically unrelated class of agents is the overexpression of drug efflux proteins such as ABC proteins. We previously examined the mRNA levels of several ABC proteins in these MCF-7 cell lines [25]. Here we quantitatively determined the expression level and localization of several ABC superfamily proteins including ABCB1, ABCC1 and ABCG2 by immunoblotting and immuofluorescence. These three ABC proteins were chosen because they are most studied clinically and are very important in breast cancer [19].

**Figure 2:**
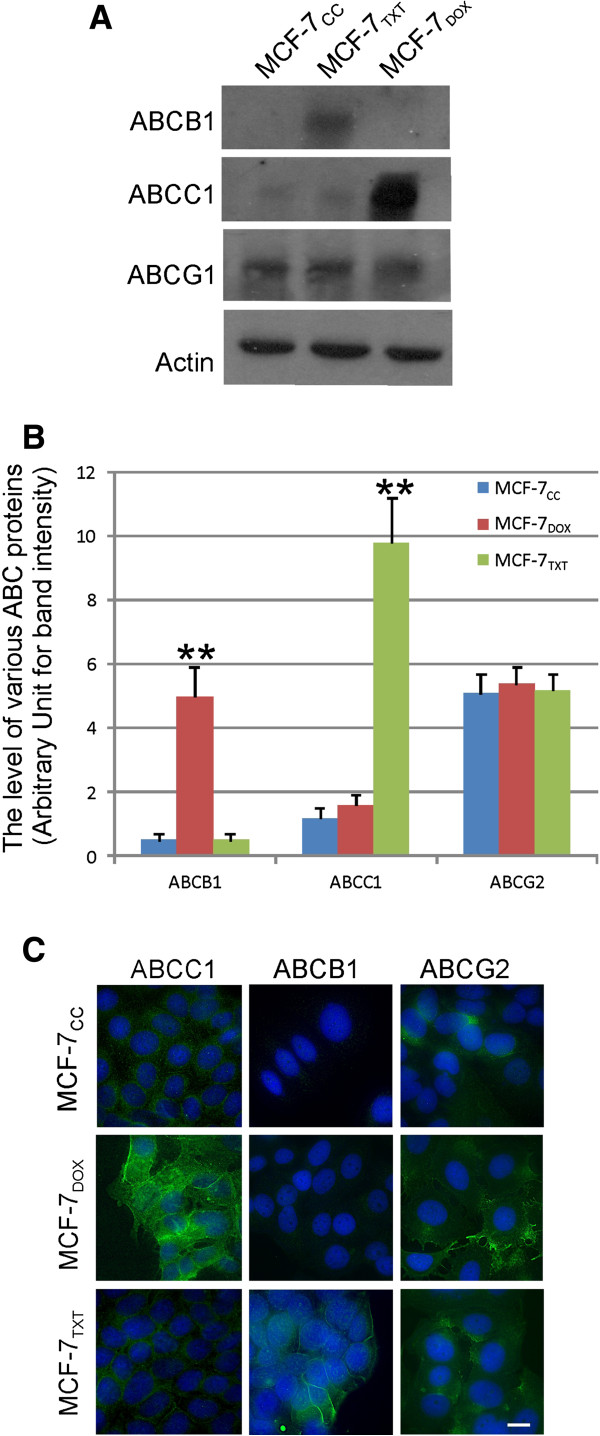
**The expression and localization of ABC proteins. (A)** The expression of various ABC proteins including ABCB1, ABCC1 and ABCG2 in MCF-7_CC_, MCF-7_TXT_ and MCF-7_DOX_ cells was determined by immunoblotting with specific antibodies. **(B)** Quantification of the data from three independent experiments as described in panel **A**. The intensity of the bands of ABC proteins was normalized against the intensity of the actin loading. The error bar is standard error. ** indicates that the difference is statistically significant with p < 0.01. **(C)** Immunofluorescence. The expression and the localization of various ABC proteins including ABCB1, ABCC1 and ABCG2 in MCF-7_CC_, MCF-7_TXT_ and MCF-7_DOX_ cells was determined by indirect immunofluorescence. The green indicates the ABC proteins and the blue is the DAPI stain for nucleus. Size bar: 10 μm.

The expression levels of ABCB1, ABCC1 and ABCG2 were first examined by immunoblotting and quantified by densitometry. As shown in Figure 2A&B, the expression level of ABCB1 was much higher in MCF-7_TXT_ cells than that in MCF-7_CC_ and MCF-7_DOX_ cells. The expression level of ABCC1 was much higher in MCF-7_DOX_ cells than that in MCF-7_CC_ and MCF-7_TXT_ cells. ABCG2 level was similar in all three cell lines, but slightly higher in MCF-7_DOX_ cells. These findings are consistent with our previous data [25].

The expression level and the localization of ABCB1, ABCC1 and ABCG2 were further examined by immunofluorescence. As in Figure 2C, ABCB1, ABCC1 and ABCG2 are all significantly localized to the plasma membrane with weak intracellular stain, consistent with their roles in drug efflux. Consistent with our immunoblotting data, MCF-7_TXT_ cells showed much stronger stain of ABCB1 than MCF-7_CC_ and MCF-7_DOX_ cells. MCF-7_DOX_ cells showed much stronger stain of ABCC1 than MCF-7_CC_ and MCF-7_TXT_ cells. The ABCG2 stain was similar in all three cell lines. These results suggest that different ABC proteins may confer resistance to different drugs.

### The effects of docetaxel treatment on the expression and subcellular localization of ABC superfamily proteins

**Figure 3:**
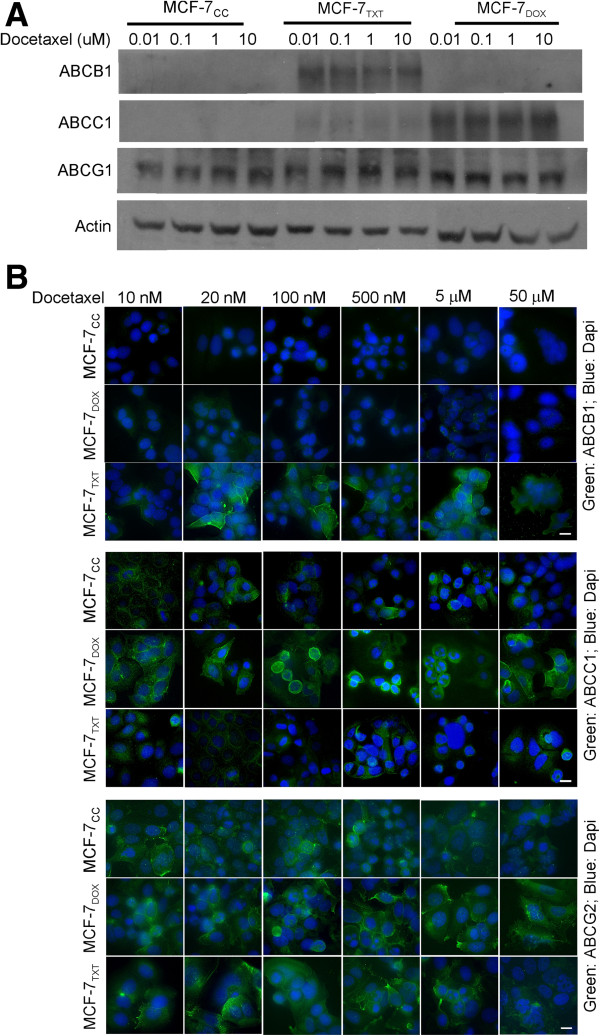
**The effects of docetaxel treatment on the expression and localization of various ABC proteins in selected MCF-7 cell lines. (A)** The effects of docetaxel on the expression of ABC proteins by immunoblotting. MCF-7_CC_, MCF-7_TXT_ and MCF-7_DOX_ cells were treated with docetaxel at indicated concentration for 24 hours. The cells were then lysed and expression level of ABCB1, ABCC1 and ABCG2 in the cells was examined by immunoblotting with specific antibodies. **(B)** The effects of docetaxel on the expression of ABC proteins by indirect immunofluorescence. MCF-7_CC_, MCF-7_TXT_ and MCF-7_DOX_ cells were seeded on coverslip and treated with docetaxel of indicated concentrations for 24 hours. The localization and expression of ABCB1, ABCC1 and ABCG2 in the cells were examined by indirect immunofluorescence. The green indicates the ABC proteins and the blue is the DAPI stain for nucleus. Size bar: 10 μm.

We next examined whether treatment of these three cell line with docetaxel changes the expression level or subcellular localization of ABCB1, ABCC1 and ABCG2 by using the same method described above. MCF-7_CC_, MCF-7_TXT_ and MCF-7_DOX_ cells were treated with docetaxel of indicated concentrations for 24 h. Western blotting showed that the expression level of ABCB1, ABCC1 and ABCG2 was little changed following the treatment (Figure 3A). Indirect immunofluorescence further showed that there was no significant change in the expression level of ABCB1, ABCC1 and ABCG2 (Figure 3B). The localization of ABCB1, ABCC1 and ABCG2 was still exclusively at the plasma membrane following docetaxel treatment (Figure 3B). We observed a stronger stain in cells arrested in M phase following docetaxel treatment. This stronger plasma membrane stain of ABCB1, ABCC1 and ABCG2 may be due to the relatively smaller size and the roundness of the cells in M phase.

### The effects of cancer drugs on apoptosis and microtubule formation

After examining the relationship between the acquired drug resistance in MCF-7_TXT_/MCF-7_DOX_ cells and the expression level of ABC proteins, we next explored the involvement of other possible mechanisms. It is well documented that taxanes kill cells by stabilizing their microtubules, which eventually causes cellular apoptosis. We, therefore, examined the effects of paclitaxel, docetaxel and doxorubicin on microtubule formation and cell apoptosis in these cells.

**Figure 4:**
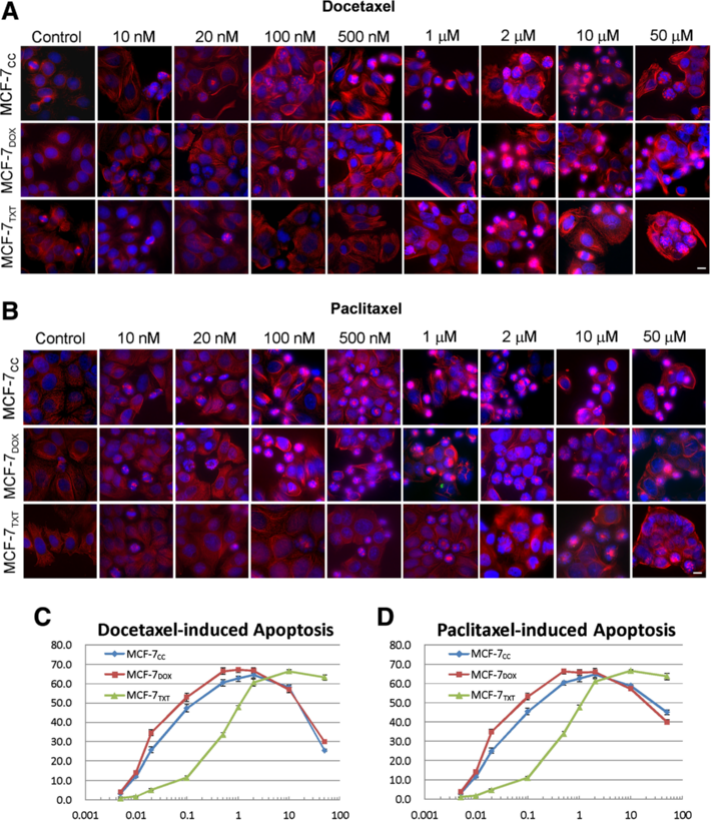
**The effects of taxanes on the microtubule formation, M-phase arrest and cell apoptosis on the selected MCF-7 cell lines. (A-B)**, MCF-7_CC_, MCF-7_TXT_ and MCF-7_DOX_ cells were seeded on coverslip and treated with docetaxel **(A)** and paclitaxel **(B)** of indicated concentrations for 24 hours. The microtubule formation, M-phase arrest and cell apoptosis were determined by fluorescence microscopy. The red indicates the α-tubulin and the blue is the DAPI stain for DNA. Size bar: 10 μm. **(C&D)** The effects of docetaxel **(C)** and paclitaxel **(D)** on the apoptosis of MCF-7_CC_, MCF-7_TXT_ and MCF-7_DOX_ cells. The apoptotic cells were determined by chromatin condensation and nuclear morphology as revealed by DAPI stain in **A** &**B**. For each data, 300 cells from at least three independent experiments were examined. The error bar is the standard error.

Microtubule formation and the cell apoptosis were examined by fluorescence microscopy (Figure 4). Microtubule formation was assayed by the tubulin stain (red). Cell apoptosis was determined by the nuclear condensation revealed by DAPI stain (blue) (Figure 4A&B). Cell apoptosis was quantitated by calculating the percentage of apoptotic cells at each given drug concentration (Figure 4C-D). Both paclitaxel and docetaxel induced much stronger cell apoptosis in MCF-7_CC_ and MCF-7_DOX_ cell than that in MCF-7_TXT_ cells at a drug concentration lower than 2 μM (Figure 4). At 10 nM, paclitaxel and docetaxel began to induce apoptosis in MCF-7_CC_ and MCF-7_DOX_ cells (10%), but a similar level of apoptosis in MCF-7_TXT_ cell only occurs when paclitaxel and docetaxel reach a concentration of 100 nM. The maximum apoptosis (approximately 60%) in MCF-7_CC_ and MCF-7_DOX_ cells was induced by paclitaxel and docetaxel at a concentration of 500 nM. Meanwhile, a similar level of apoptosis in MCF-7_TXT_ cell was induced by paclitaxel and docetaxel at a concentration of 10 μM. However, at a concentration equal or above 2 μM, paclitaxel and docetaxel caused a similar level of apoptosis in all three cell lines. More interestingly, the percentage of apoptotic cells actually decreased at drug concentrations higher than 2 μM in all three cell lines. These results indicate that the resistance of MCF-7_TXT_ cells to paclitaxel/docetaxel is due to their resistance to apoptosis induced by paclitaxel/docetaxel.

It is clear that paclitaxel/docetaxel induce cell apoptosis by stabilizing the microtubule spindle, arresting the cell at M phase. We next examined whether the resistance to paclitaxel/docetaxel-induced apoptosis in MCF-7_TXT_ cells is due to resistance to drug-induced tubulin polymerization. As shown in Figure 4A&B, in the absence of paclitaxel/docetaxel, cells go through the cell cycle with the formation of normal tubulin spindle in M phase (Figure 4A&B, Control). However, at 10 nM, paclitaxel/docetaxel begins to arrest MCF-7 and MCF-7_DOX_ cells in M phase by interfering with the assembling/disassembling microtubule spindle, which leads to cell apoptosis as revealed by nuclear condensation. However, in MCF-7_TXT_ cells, 10 nM of paclitaxel/docetaxel did not interfere with microtubule spindle assembling/disassembling, which was revealed by the presence of mitotic cells with normal microtubule spindles. For MCF-7_CC_ and MCF-7_DOX_ cells, the increase in concentration of paclitaxel/docetaxel from 10 nM to 500 nM caused an increased prevalence of M phase arrest by forming abnormal mitotic spindles. On the other hand, it also stimulated strong microtubule formation for cells in interphase as revealed by the thick microtubule fibers throughout the cell. For the same range of paclitaxel/docetaxel concentration, we observed few abnormal mitotic spindles and did not observe any significant change in the microtubule formation in MCF-7_TXT_ cells. With a further increase of the concentration from 500 nM to 10 μM, more MCF-7_TXT_ cells were arrested at M phase with abnormal mitotic spindles and the formation of microtubule fibres was strongly enhanced in interphase cells. For MCF-7_CC_ and MCF-7_DOX_ cells, the percentages of the cells arrested at M phase with abnormal mitotic spindles were not changed significantly with the increase of the drug concentration from 500 nM to 10 μM, but microtubule formation in interphase cells was further enhanced. At a concentration of 50 μM, fewer cells were arrested at M phase with abnormal mitotic spindles, but microtubule fibres were thicker and formed circles at the cell periphery in both MCF-7_CC_ and MCF-7_DOX_ cells. Similar effects were observed in MCF-7_TXT_ cells, but to a lesser degree (Figure 4A&B).

Together, these results indicate that at a concentration as high as 500 nM, paclitaxel/docetaxel do not cause significant formation of abnormal mitotic spindles and abnormal microtubule fibres in the taxane-resistant MCF-7_TXT_ cells. The resistance of MCF-7_TXT_ cell to paclitaxel/docetaxel is likely due to the insensitivity of its microtubule system to the drugs.

**Figure 5:**
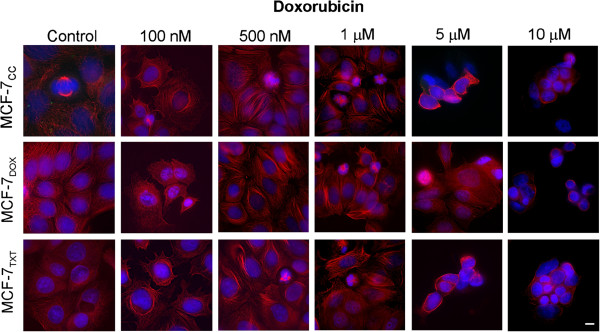
**The effects of doxorubicin on the microtubule formation, M-phase arrest and cell apoptosis on the selected MCF-7 cell lines.** MCF-7_CC_, MCF-7_TXT_ and MCF-7_DOX_ cells were seeded on coverslip and treated with doxorubicin of indicated concentrations for 24 hours. The microtubule formation, M-phase arrest and cell apoptosis were determined by fluorescence microscopy. The red indicates the α-tubulin and the blue is the DAPI stain for DNA. Size bar: 10 μm.

We also examine the effects of doxorubicin on the apoptosis and microtubule formation. As shown in Figure 5, very few cells were arrested at the M phase with an increased concentration of doxorubicin. Very few cells showed condensed chromatin and fragmented nuclei even at high doxorubicin concentrations. Very few cells were left on the coverslips when the doxorubicin concentration was above 1 μM in MCF-7_CC_ and MCF-7_TXT_ cells, or when the concentration was above 5 μM in MCF-7_DOX_ cells. We have shown in Figure 1 that at 1 μM, more than 50% of the MCF-7_CC_ and MCF-7_TXT_ cell were killed by doxorubicin, and at 5 μM, more than 50% of the MCF-7_DOX_ cells were killed by doxorubicin. Thus, it seemed that the dead cells detached from the coverslip following their treatment with doxorubicin.

### Live imaging of chromatin condensation induced by docetaxel

**Figure 6:**
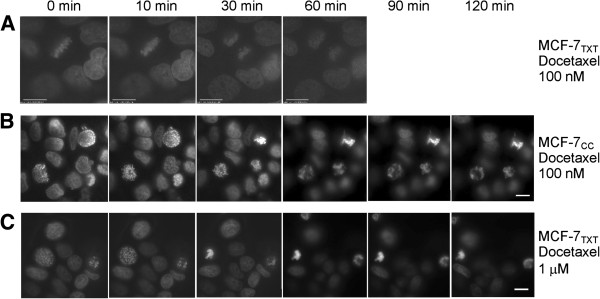
**The effects of docetaxel on the M-phase arrest in MCF-7**_**CC **_**and MCF-7**_**TXT **_**cells.** The effects of docetaxel on the chromosome condensation and M-phase arrest of MCF-7_CC_ and MCF-7_TXT_ cells were examined by live imaging. MCF-7 cells were incubated with DMEM containing 250 ng/ml Hoechst 33342 (Calbiochem) for 10 minutes to stain DNA. Then, the cells were incubated with docetaxel of indicated concentration. The images of cell mitosis were recorded every 2 minutes by live imaging. **(A)** MCF-7TXT cells treated with 100 nM Docetaxel. **(B)** MCF-7CC cells treated with 100 nM Docetaxel. **(C)** MCF-7TXT cells treated with 1 μM Docetaxel. The Size bar: 10 μm.

We next employed a live imaging approach to examine the effects of docetaxel on chromatin condensation (an indicator for cell apoptosis). As shown in Figure 6A and Additional file 1: Video S1, at a concentration of 20 nM, docetaxel had no noticeable effects on the progression of mitosis in MCF-7_TXT_ cells. The cell cycle smoothly passed through M phase with normal chromosome pairing and segregation. However, for MCF-7_CC_ cells, the same concentration of docetaxel disrupted the normal movement of chromosomes at M phase and resulted in M-phase arrest, leading to chromatin condensation (Figure 6B, Additional file 1: Video S2A&B). For MCF-7_TXT_ cells, a concentration of 500 nM is needed for docetaxel to disrupt chromosome movement during M phase and arrest the cells at M phase with condensed chromatin (Figure 6C and Additional file 1: Video S3A&B).

### The effects of docetaxel on microtubule dynamics of MCF-7_TXT_ cells

**Figure 7:**
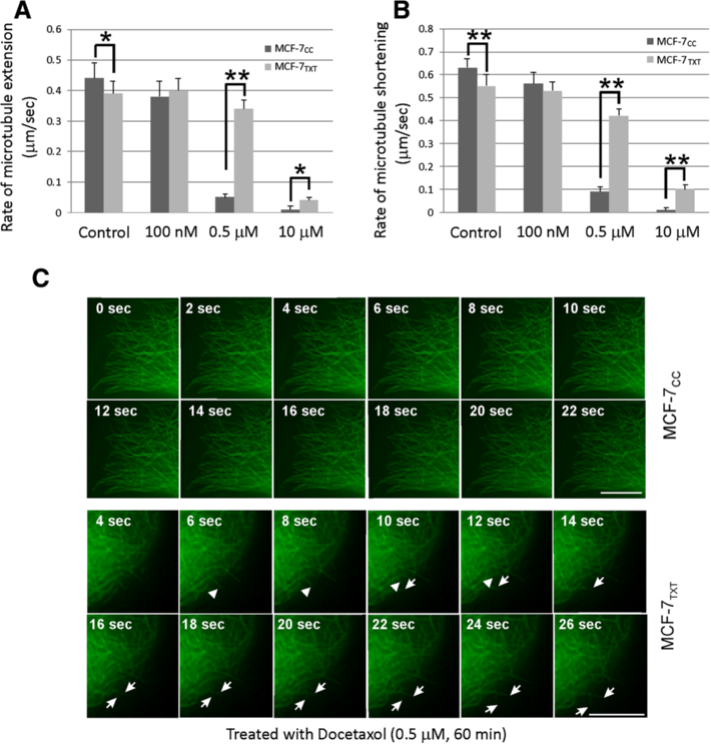
**The effects of docetaxel on the microtubule dynamics of MCF-7**_**TXT **_**an MCF-7**_**CC **_**cells.** The Live imaging was performed as described in Methods. Following the transfection of the cells with GFP-tagged α-tubulin for 24 hours, the cells were incubated with docetaxel of indicated concentration for 1 hour. The images of microtubule dynamics of MCF-7_CC_ and MCF-7_TXT_ cells were recorded every 2 seconds by live imaging. The extension rate **(A)** and shortening rate **(B)** of microtubules were measured from the recorded images. Each datum is the average of 20 measurements from at least 8 different cells. The error bar is the standard error. * indicates that the difference is statistically significant with p < 0.05. ** indicates that the difference is statistically significant with p < 0.01. **(C)** Selected images from the live imaging (Additional file 3: Video S8&9) of microtubule dynamics of MCF-7_CC_ and MCF-7_TXT_ cells following treatment with 0.5 μM docetaxel for 1 hour. Arrow indicates the extending microtubules. Arrow head indicates the shortening microtubules. Size bar, 10 μm.

To further determine whether the resistance of MCF-7_TXT_ cells to docetaxel is related to the insensitivity of its microtubule polymerisation/depolymerisation to the drugs, we examined the effects of docetaxel on microtubule dynamics by live imaging (time-lapse microscopy). We transiently expressed GFP-tubulin in MCF-7_TXT_ and MCF-7_CC_ cells for 24 h. At the flat lamellar edge of interphase cells, GFP-labeled microtubules are readily visible and can be followed by time-lapse fluorescence microscopy. Microtubule dynamics were measured by the extending and shortening of the microtubules and expressed as μm/sec. As shown in Figure 7, in the absence of docetaxel treatment, the microtubules were more dynamic in MCF-7_CC_ cells than in MCF-7_TXT_ cells. Both the extending rate and the shortening rate of microtubules in MCF-7_TXT_ cells were significantly lower than those in MCF-7_CC_ cells (Figure 7A&B, Additional file 2: Figure S1, Additional file 3: Video S4&5). However, the microtubule dynamics in MCF-7_TXT_ cells were much less sensitive to docetaxel than those in MCF-7_CC_ cells. At 100 nM of docetaxel, both the extending rate and the shortening rate of microtubules were slightly decreased in MCF-7_CC_ cells, but not in MCF-7_TXT_ cells (Figure 7A&B, Additional file 4: Figure S2, Additional file 3: Video S6&7). At a concentration of 0.5 μM, while both the extending rate and the shortening rate of microtubules were greatly decreased in MCF-7_CC_ cells, microtubule dynamics were only slightly inhibited in MCF-7_TXT_ cells (Figure 7A, B&C, Additional file 3: Video S8&9). At 10 μM docetaxel, the microtubules in both MCF-7_CC_ and MCF-7_TXT_ cells were almost completely stabilized with very low extending and shortening rate (Figure 7A&B, Additional file 5: Figure S3, Additional file 3: Video S10&11). These results indicate that MCF-7_TXT_ cells may have less vigorous intrinsic microtubule dynamics than MCF-7_CC_ cells. The microtubule dynamics in MCF-7_TXT_ cells were ten times less sensitive to docetaxel treatment. This insensitivity to docetaxel contributes to the resistance of MCF-7_TXT_ cells to docetaxel.

### The expression levels of β-tubulin isoforms in MCF-7_TXT_, MCF-7_DOX_, and MCF-7_CC_ cells

**Figure 8:**
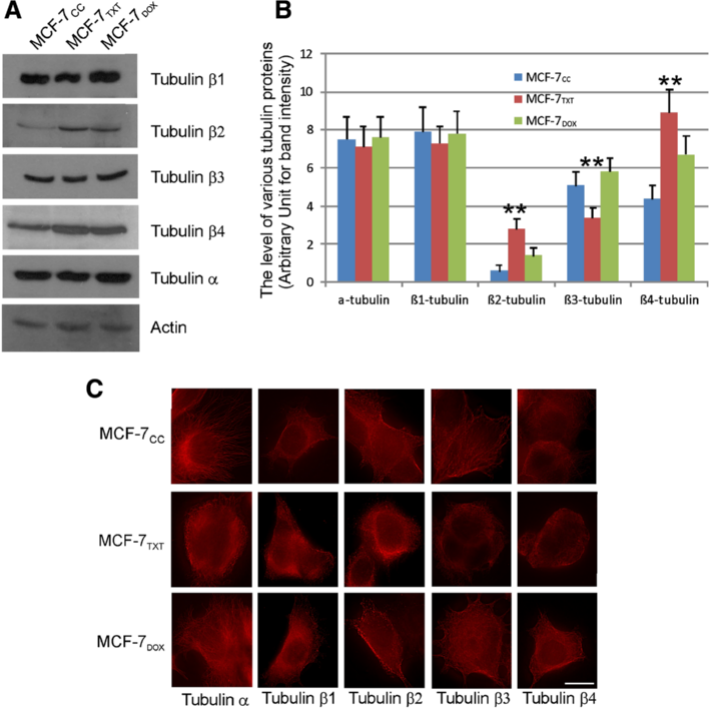
**The expression and localization of various β-tubulin isoforms in selected MCF-7 cells. (A)** The expression of α-tubulin and various β-tubulin isoforms including β-1, β2, β3, and β-4 tubulin in MCF-7_CC_, MCF-7_TXT_ and MCF-7_DOX_ cells was determined by immunoblotting with specific antibodies. **(B)** Quantification of the data from three independent experiments as described in panel **A**. The intensity of the bands of various tubulin proteins was normalized against the intensity of the actin loading. The error bar is standard error. ** indicates that the difference is statistically significant with p < 0.01. **(C)** Immunofluorescence. The expression and the localization of various tubulin proteins including α-, β1-, β2-, β3-, and β4- tubulin in MCF-7_CC_, MCF-7_TXT_ and MCF-7_DOX_ cells was determined by indirect immunofluorescence. The red indicates the various tubulin proteins. Size bar: 10 μm.

We next examined the expression level of various β-tubulin isoforms because it has been reported that the microtubule dynamics may be related to the expression level of various β-tubulin isoforms [3,4,14]. The expression of α-tubulin and various β-tubulin isoforms including β1-, β2-, β3- and β4-tubulin was examined in MCF-7_TXT_, MCF-7_DOX_ and MCF-7_TXT_ cells by immunoblotting and immunofluorescence. As shown in Figure 8A, the expression levels of α-tubulin and β1-tubulin were high and very similar in the three MCF-7 cell lines. However, the expression levels of other β-tubulins were different. The expression level of β3-tubulin in MCF-7_TXT_ cells was significantly lower than that in MCF-7_CC_ and MCF-7_DOX_ cells (p < 0.01). On the other hand, the expression levels of β2- and β4-tubulin in MCF-7_TXT_ cells were significantly higher than that in MCF-7_CC_ and MCF-7_DOX_ cells (p < 0.01).

We also examined the expression and localization of the α-tubulin and various β-tubulin isoforms in MCF-7_CC_, MCF-7_TXT_ and MCF-7_DOX_ cells by indirect immunofluorescence (Figure 8B). As shown in Figure 8B, α-tubulin stain showed clear and intact microtubules in all three cell lines. The β3- and β4-tubulins stain also showed clear and intact microtubules in both MCF-7_CC_ and MCF-7_DOX_ cells, but to a lesser extent in MCF-7_TXT_ cells. However, staining of the β1- and β2-tubulin did not appear like microtubules, but rather small dots filled the cytoplasm of the cells. The pattern of these small tubulin dots is more defined and localized discontinuously along the microtubules in both MCF-7_CC_ and MCF-7_DOX_ cells. However, the patterns of these small tubulin dots are messy and less defined along the microtubule fibers in MCF-7_TXT_ cells. Another difference between MCF-7_TXT_ cell and MCF-7_CC_/MCF-7_DOX_ cell is that in MCF-7_TXT_ cells, the β1- and β2-tubulin are localized more at the perinuclear region of the cell while the β3- and β4-tubulin were localized more at the peripheral region of the cell.

Together, our results showed that the expression and localization of the various β-tubulin isoforms were significantly altered in MCF-7_TXT_ cells. It is likely that the difference in the expression level of various β-tubulin isoforms and their subcellular localization between MCF-7_TXT_ cells and MCF-7_CC_/MCF-7_DOX_ cells contributes to the differences in their observed microtubule dynamics and resistance to taxanes.

## Discussion and conclusion

To better understand the mechanisms underlying acquired resistance to taxanes in breast cancer, we utilized previously established cell lines in which MCF-7 breast cancer cells were selected for survival in increasing concentrations of doxorubicin (MCF-7_DOX_ cells) or docetaxel (MCF-7_TXT_ cells) [25]. A cell line selected under identical conditions in the absence of drug (MCF-7_CC_) was used as a control.

We showed that MCF-7_TXT_ cells that are resistant to docetaxel are cross resistant to other drugs in the same group such as paclitaxel, but are not resistant to doxorubicin, a different type of cancer drug (Figure 1). Similarly, we showed that MCF-7_DOX_ cells are resistant to doxorubicin, but not resistant to both docetaxel and paclitaxel. These results demonstrate that the acquired chemoresistance in this instance is specific to the selection agent and it is not a consequence of the establishment of mechanisms of multidrug resistance. Our finding is different from a previous report showing that drug resistant MCF-7 cells lines also develops cross-resistance to structurally unrelated cancer drugs [27]. However, in this previous report, it is also shown that selected paclitaxel-resistant MCF-7 cell is not cross-resistant to doxorubicin, which is consistent to our data. Nevertheless, our data suggest that the acquired resistance can be specific and chemotherapy using combined drugs or alternative drugs may overcome the resistance. Indeed, sequential single-agent therapy or combination therapy have been used in breast cancer treatment to overcome drug resistance [28].

We further showed that the selected chemoresistant cell lines do have higher expression level of certain ABC transporter proteins (Figure 2). The expression level of ABCB1 is very high only in MCF-7_TXT_ cells and the expression level of ABCC1 is very high only in MCF-7_DOX_ cells. The expression level of ABCG2 is similar in both the selected chemoresistant and the parental MCF-7 cell lines and likely did not play a role in the drug-resistant phenotypes of these cell lines. These observations are consistent with our previous findings regarding the transcription of these ABC transporter genes in various selected MCF-7 cells [25]. We also showed previously that MCF-7_TXT_ cells have lower accumulation of paclitaxel and MCF-7_DOX_ cells have lower accumulation of doxorubicin [25]. While all of these three ABC proteins have been implicated in multiple drug resistance including taxanes and doxorubicin [1,3,4,14,29], our results suggest that the specific member of ABC transporter proteins that are induced during the selection process may be different depending upon the selection agent. Our results indicate that the resistance to taxanes in MCF-7_TXT_ cells is associated with high expression level of the ABCB1 protein, but not ABCC1 and ABCG2, which is consistent with previous findings in other cell lines [19]. Although ABCB1, ABCC1 and ABCG2 are all implicated in the resistance to doxorubicin [19], the lack of cross-resistance to doxorubicin in MCF-7_TXT_ cells suggests that the ABCB1 overexpression alone may not efficiently mediate the efflux of doxorubicin in this selected MCF-7 cell line. Moreover, the overexpression of ABCC1, but not ABCB1 in MCF-7_DOX_ cells suggests that the resistance to doxorubicin is associated with high expression of the ABCC1 protein and lack of overexpression of ABCB1 does not block the ability of cells to acquire the resistance to doxorubicin. The lack of cross-resistance to taxanes in MCF-7_DOX_ cells also support the previous finding that ABCC1 may be more efficient in mediating the efflux of doxorubicin, but not taxane [19,28]. Given that the expression of ABCG2 protein was similar amongst MCF-7CC, MCF-7TXT, and MCF-7DOX cells, our data also suggest that resistance to either taxanes or doxorubicin is unrelated to ABCG2 expression. Our observation that treatment of the above cell lines with docetaxel for 24 h did not alter the expression and localization of these ABC proteins (Figure 3) also suggests that docetaxel itself cannot induce the expression of ABC transporter proteins.

It is also likely that the overexpression of any one of the above ABC proteins is not sufficient to confer the resistance to chemotherapy and other mechanisms are responsible for the resistance. Indeed, as we showed previously, while the drug resistance is related to the expression of drug transporters and the drug accumulations in the cells, drug transporter inhibitors are insufficient to fully restore sensitivity of MCF-7_DOX_ cells to doxorubicin or MCF-7_TXT_ cells to docetaxel [25]. A series of experiments are performed in this study to provide insight into the additional mechanisms underlying resistance to taxanes in these cells.

We show in this study that the acquired resistance of MCF-7_TXT_ cells to taxanes as revealed in cytotoxicity assay (Figure 1) is also associated with resistance to taxane-induced apoptosis as revealed by quantification of chromosome condensation (Figure 4). While some studies suggest that MCF-7 cells are unable to go apoptosis due to the deletion of caspase-3 gene and thus the lack of caspase-3 protein, other studies indicate that MCF-7 cells are able to go apoptosis due to the existence of caspase-3 independent apoptotic pathways [30-36]. We found in the cytotoxicity assay that the total of surviving cells follow a two-phase decrease with the increase of the concentration of docetaxel and paclitaxel (Figure 1). A sharp decrease at the low dose range (< 1 μM) and a further decrease at the high dose rage (> 10 μM) following a plateau at the middle dose range (1-10 μM). This observation is consistent with our previous report [37]. We further showed in this study that taxane treatment arrested the cells at M phase at the dosage lower than 1 μM, which eventually leads to cell apoptosis (Figure 4A). The first phase of decrease in cell population (Figure 1) is coincident with the increase of cell apoptosis induced by the treatment of docetaxel and paclitaxel (Figure 4), which suggest that taxane-induced apoptosis is likely responsible for the reduction of cell population at the doses less than 1 μM. On the other hand, treatment of the cells with doxorubicin did not arrest the cells at M phase and did not induce significant cell apoptosis (Figure 5). It has been reported that doxorubicin interacts with topoisomerases I and II [10] leading to DNA damage followed by G1 and G2 growth arrest, which has been proposed to correlate with tumor response and patient’s outcome [1,11]. The few apoptotic cells induced by doxorubicin observed under a fluorescence microscope could be due to the detaching of the apoptotic cells from the coverslip.

The interaction between taxanes and the microtubules stabilises microtubules by reducing depolymerisation. Stabilization of microtubules by taxane binding prevents normal formation of mitotic spindles [6]. This leads to chronic activation of the spindle assembly checkpoint (SAC), which in turn leads to mitotic arrest [7]. Extended mitotic arrest eventually leads to cell death [8]. We studied the effects of taxanes on the formation of microtubules and the mitotic spindles in MCF-7_TXT_, MCF-7_DOX_ and MCF-7_CC_ cells by indirect immunofluorescence (Figure 4A). We showed that the abnormal mitotic spindles induced by taxane treatment are accompanied by the arrest of cells at M phase and the initiation of cell apoptosis (nuclear condensation). While 10 - 100 nM of docetaxel and paclitaxel induced significant mitotic spindle disruption and M phase arrest in MCF-7_CC_ and MCF-7_DOX_ cells, ten times higher concentrations of docetaxel and paclitaxel are needed to induce a similar level of mitotic spindle disruption and M phase arrest in MCF-7_TXT_ cells. Thus, our data clearly suggests that the acquired resistance to taxanes in MCF-7_TXT_ cells is due to the resistance to taxane-induced mitotic spindle disruption and M phase arrest.

We also examined microtubule dynamics in MCF-7_TXT_ cells (Figure 7, Additional file 2: Figure S1, Additional file 4: Figure S2, Additional file 5: Figure S3 and Additional file 3: Video S4-10). We showed that in the absence of docetaxel treatment the microtubule dynamics are robust in both MCF-7_TXT_ and MCF-7_CC_ cells, but the microtubule dynamics are weaker in MCF-7_TXT_ cells than that in MCF-7_CC_ cells. Moreover, microtubule dynamics are greatly more insensitive to docetaxel in MCF-7_TXT_ calls than in MCF-7_CC_ cells. For example, treatment with 0.5 μM docetaxel only slightly reduces the microtubule dynamics in MCF-7_TXT_ cells, but significantly reduced both the shortening and extending rate of microtubules in MCF-7_CC_ cells. Our findings suggest that the resistant MCF-7_TXT_ cells have unique microtubule dynamics that are likely unrelated to the overexpression of ABC transporters. The insensitivity of microtubules to docetaxel treatment in MCF-7_TXT_ cells may be partially the reason that docetaxel is less effective in inducing the M-phase arrest and the apoptosis in MCF-7_TXT_ cells in comparison to MCF-7_CC_ cells. This unique microtubule dynamics may contribute to the resistance to docetaxel.

Although taxane-binding sites on microtubules are only present in assembled tubulin [38], the stabilized microtubules are not able to extend without depolymerisation. It is interesting to note that 0.5 μM docetaxel induces very high level M-phase arrest at MCF-7_TXT_ cells (Figures 1 and 4), but does not significantly reduce the microtubule dynamics in MCF-7_TXT_ cells (Figure 7). Similarly, 100 nM of docetaxel induces very high level M-phase arrest at MCF-7_CC_ cells (Figures 1 and 4), but does not significantly reduce the microtubule dynamics in MCF-7_CC_ cells (Figure 7). The reason for this could be the duration of the treatment. We assayed the M-phase arrest and cell apoptosis following the treatment for 24 h, but we assayed the microtubule dynamics only after the treatment for 1 h. Besides, multiple mechanisms are involved in the acquired resistance to docetaxel, microtubule dynamics is just one of these mechanisms.

Finally, we showed that the all four β-tubulin isoforms are expressed in the three MCF-7 cell lines. While the relative expression levels of the four β-tubulin isoforms are very similar between MCF-7_DOX_ and MCF-7_CC_ cells, the relative expression levels of the β-tubulin isoforms are quite different in MCF-7_TXT_ cells (Figure 8). MCF-7_TXT_ cells have relatively higher β2- and β4-tubulin expression and relatively lower β3-tubulin expression level (Figure 8). These results suggest that the expression level of various β-tubulin isoforms is related to the microtubule dynamics of the MCF-7 cells in response to docetaxel treatment. The expression levels of various tubulin isoforms have been linked to the resistance to taxanes in breast cancers. It has been reported that both β3- and β4-tubulin are overexpressed in a MCF-7 cell line selected for resistant to paclitaxel under increased paclitaxel concentration [21]. The overexpression of β3-tubulin induces paclitaxel resistance by reducing the ability of paclitaxel to suppress microtubule dynamics [20]. It is also reported that mRNA levels of β2-, β3- and β4-tubulin are significantly upregulated in paclitaxel- and docetaxel-resistant MCF-7 cells [23]. The MCF-7_TXT_ cell line used in this research is selected for resistance to docetaxel, but showed similar resistance to paclitaxel. While we also found that MCF-7_TXT_ cells have higher β2- and β4-tubulin expression than MCF-7_CC_ and MCF-7_DOX_ cells, we actually showed that the β3-tubulin expression level is lower. The significant difference in the expression levels of various β-tubulin isoforms suggest that the composition of β-tubulin in the formation of microtubules may contribute to the microtubule dynamics and its response to taxane treatment, which could be part of the mechanisms underlying the acquired resistance to taxanes in breast cancer cells.

We also examined the localization of these tubulin isoforms. We showed that the localization pattern of the various β-tubulin isoforms in MCF-7_TXT_ cells is different from that of MCF-7_CC_ and MCF-7_DOX_ cells (Figure 8B). While we did not know how the different subcellular distribution of these β-tubulin isoforms affects its response to docetaxel treatment, it is possible that the relative composition of various β-tubulin isoforms in microtubules and their formation pattern may play a role in determining microtubule dynamics and sensitivity of microtubules to docetaxel treatment.

As shown from this study, multiple mechanisms are likely involved in the acquired drug resistance. Besides discussed above, many other proteins and mechanisms may also be involved in the acquired drug resistance. It has been shown that extracellular matrix proteins, apoptosis related proteins, cytokine and growth factor signaling proteins, and many other proteins are overexpressed in the selected MCF-7 cells resistant to taxanes and other cancer drugs [29,39,40]. It is interesting to find out in the future study whether and how these different mechanisms regulate drug-resistance independently or coordinately.

In conclusion, our results suggest the presence of multiple mechanisms for acquired drug resistance to taxanes. Prolonged exposure to taxanes may result in the selection of the breast cancer cells that overexpress certain drug resistance proteins, such as ABCB1 in MCF-7_TXT_ cells, which will lower the taxane level inside the cells and thus contribute to the resistance to taxanes. Prolonged exposure to taxanes may also result in the selection of the breast cancer cells that have differential expression of various β-tubulin isoforms, such as higher β-2 and β-4 and lower β-3 tubulin in MCF-7_TXT_ cells. In addition, the relative composition of various β-tubulin isoforms within the microtubules and the specific distribution of these β-tubulin isoforms along the microtubules may determine the dynamics of the microtubules and its sensitivity to taxane treatment. For example, in MCF-7_TXT_ cells, the distinct distribution of the β-tubulin isoforms can be related to the weak microtubule dynamics and its insensitivity to taxane treatment.

## Competing interests

All authors declare that they have no competing interests.

## Authors’ contributions

**HW**: Participating in the design of the project, performing most of the experiments including cell culture, cytotoxicity assay, immunoblotting, immunofluorescence, and living image, and participating in the data analysis and manuscript writing. **TV**: Performing experiments including cell culture and immunoblotting, and participating in data analysis. **AH**: Performing experiments including cell culture, cytotoxicity assay, and immunoblotting, and participating in data analysis. **SL**: Performing experiments including cell culture, cytotoxicity assay, and immunoblotting, and participating in data analysis. **XC**: Performing experiments including cell culture, cytotoxicity assay, immunoblotting, immunofluorescence, and living image, and participating in data analysis. **AMP**: Providing drug-resistance cell lines, participating in the design of the project, and participating in the writing of the manuscript. **DNB**: Participating in the design of the project, data analysis and the writing of the manuscript. **ZW**: Participating in the design of the project, performing some experiments including living image and immunofluorescence, and participating in data analysis and the writing of the manuscript. All authors read and approved the final manuscript.

## Pre-publication history

The pre-publication history for this paper can be accessed here:

http://www.biomedcentral.com/1471-2407/14/37/prepub

## Supplementary Material

Additional file 1: Video S1-S3 Live imaging of docetaxel-induced M-phase arrest in MCF-7wt and MCF-7txt cells. MCF-7 cells were incubated with DMEM containing 250 ng/ml Hoechst 33342 (Calbiochem) for 10 minutes to stain DNA. Then, the cells were incubated with docetaxel of indicated concentration. The images of cell mitosis were recorded every 2 minutes by live imaging. **Video S1.** MCF-7txt treated with 100 nM docetaxel. **Video S2A&B.** MCF-7wt cells treated with 100 nM docetaxel. **Video S3A&B**, MCF-7wt cells treated with 1 μM docetaxel. Click here for file

Additional file 2: Figure S1 Selected images from the live imaging (Additional file 3: Video S4&5) of microtubule dynamics of MCF-7wt **(A)** and MCF-7txt **(B)** cells without docetaxel treatment. Arrow indicates the extending microtubules. Arrow head indicates the shortening microtubules. Size bar, 10 μm. Click here for file

Additional file 3: Video S4-S11 Live imaging of the micrtotubule dynamics of MCF-7wt and MCF-7txt cells following the treatment with docetaxel. The Live imaging was performed as described in Methods. Following the transfection of the cells with GFP-tagged α-tubulin for 24 hours, the cells were incubated with docetaxel of indicated concentration for 1 hour. The images of microtubule dynamics of MCF-7wt and MCF-7txt cells were recorded every 2 seconds by live imaging. **Video S4.** MCF-7wt cells without docetaxel treatment (Control). **Video S5.** MCF-7txt cells without docetaxel treatment (Control). **Video S6.** MCF-7wt cells treated with 100 nM docetaxel for 1 hour. **Video S7.** MCF-7txt cells treated with 100 nM docetaxel for 1 hour. **Video S8.** MCF-7wt cells treated with 0.5 μM docetaxel for 1 hour. **Video S9.** MCF-7txt cells treated with 0.5 μM docetaxel for 1 hour. **Video S10.** MCF-7wt cells treated with 10 μM docetaxel for 1 hour. **Video S11.** MCF-7txt cells treated with 10 μM docetaxel for 1 hour. Click here for file

Additional file 4: Figure S2 Selected images from the live imaging (Additional file 3: Video S6&7) of microtubule dynamics of MCF-7wt **(A)** and MCF-7txt **(B)** cells following treatment with 100 M docetaxel for 1 hour. Arrow indicates the extending microtubules. Arrow head indicates the shortening microtubules. Size bar, 10 μm. Click here for file

Additional file 5: Figure S3 Selected images from the live imaging (Additional file 3: Video S6&7) of microtubule dynamics of MCF-7wt **(A)** and MCF-7txt **(B)** cells following treatment with 10 μM docetaxel for 1 hour. Arrow indicates the extending microtubules. Arrow head indicates the shortening microtubules. Size bar, 10 μm. Click here for file
