# Supplementary figures and images for: Multiple mechanisms underlying acquired resistance to taxanes in selected docetaxel-resistant MCF-7 breast cancer cells
Source: BMC Cancer. 2014 Jan 22;14:37. doi: 10.1186/1471-2407-14-37 (PMC3900991; doi:10.1186/1471-2407-14-37)

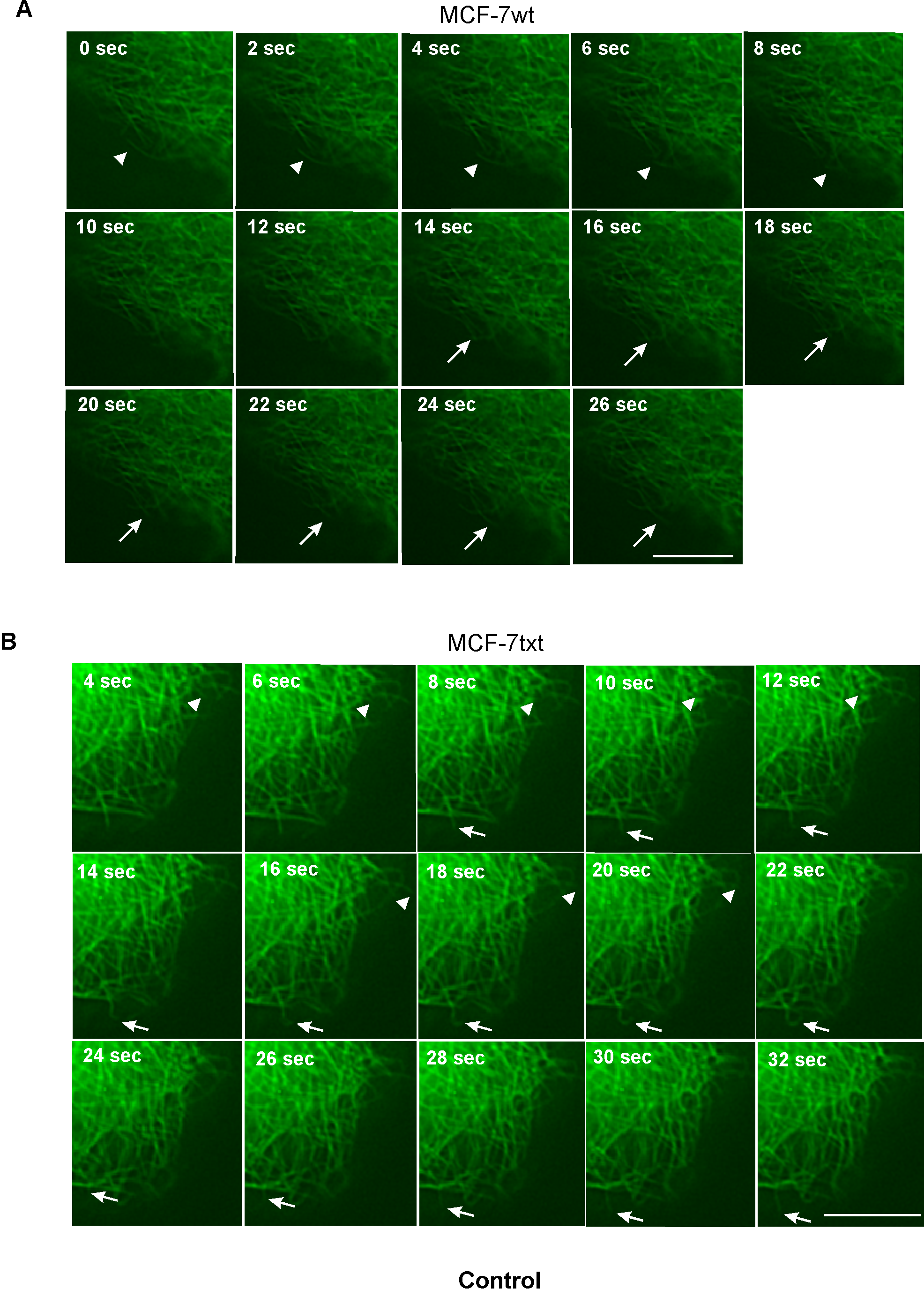

Supplement: Additional file 2: Figure S1 — Selected images from the live imaging (Additional file 3: Video S4&5) of microtubule dynamics of MCF-7wt (A) and MCF-7txt (B) cells without docetaxel treatment. Arrow indicates the extending microtubules. Arrow head indicates the shortening microtubules. Size bar, 10 μm. [file 1471-2407-14-37-S2.tiff]

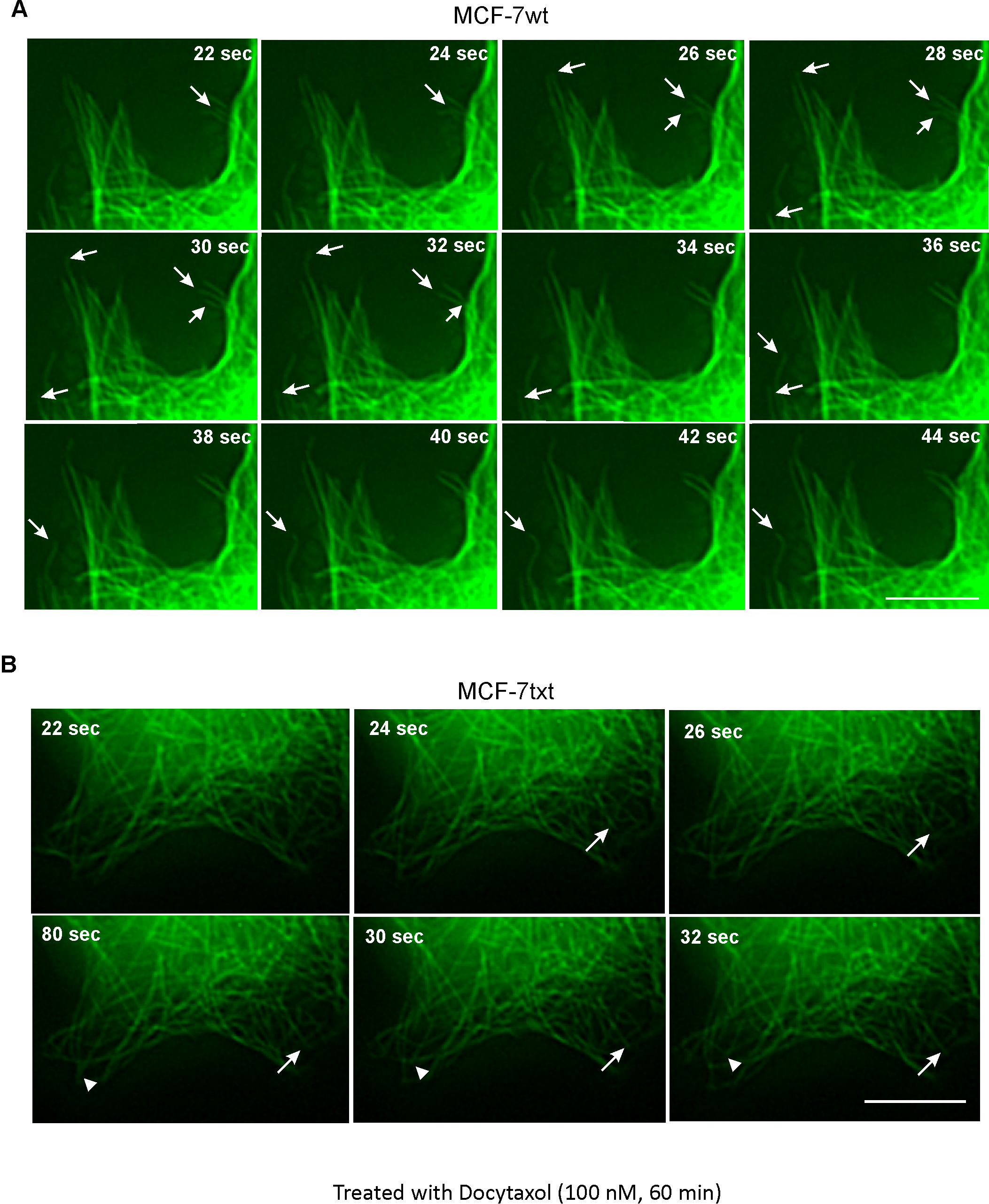

Supplement: Additional file 4: Figure S2 — Selected images from the live imaging (Additional file 3: Video S6&7) of microtubule dynamics of MCF-7wt (A) and MCF-7txt (B) cells following treatment with 100 M docetaxel for 1 hour. Arrow indicates the extending microtubules. Arrow head indicates the shortening microtubules. Size bar, 10 μm. [file 1471-2407-14-37-S4.tiff]

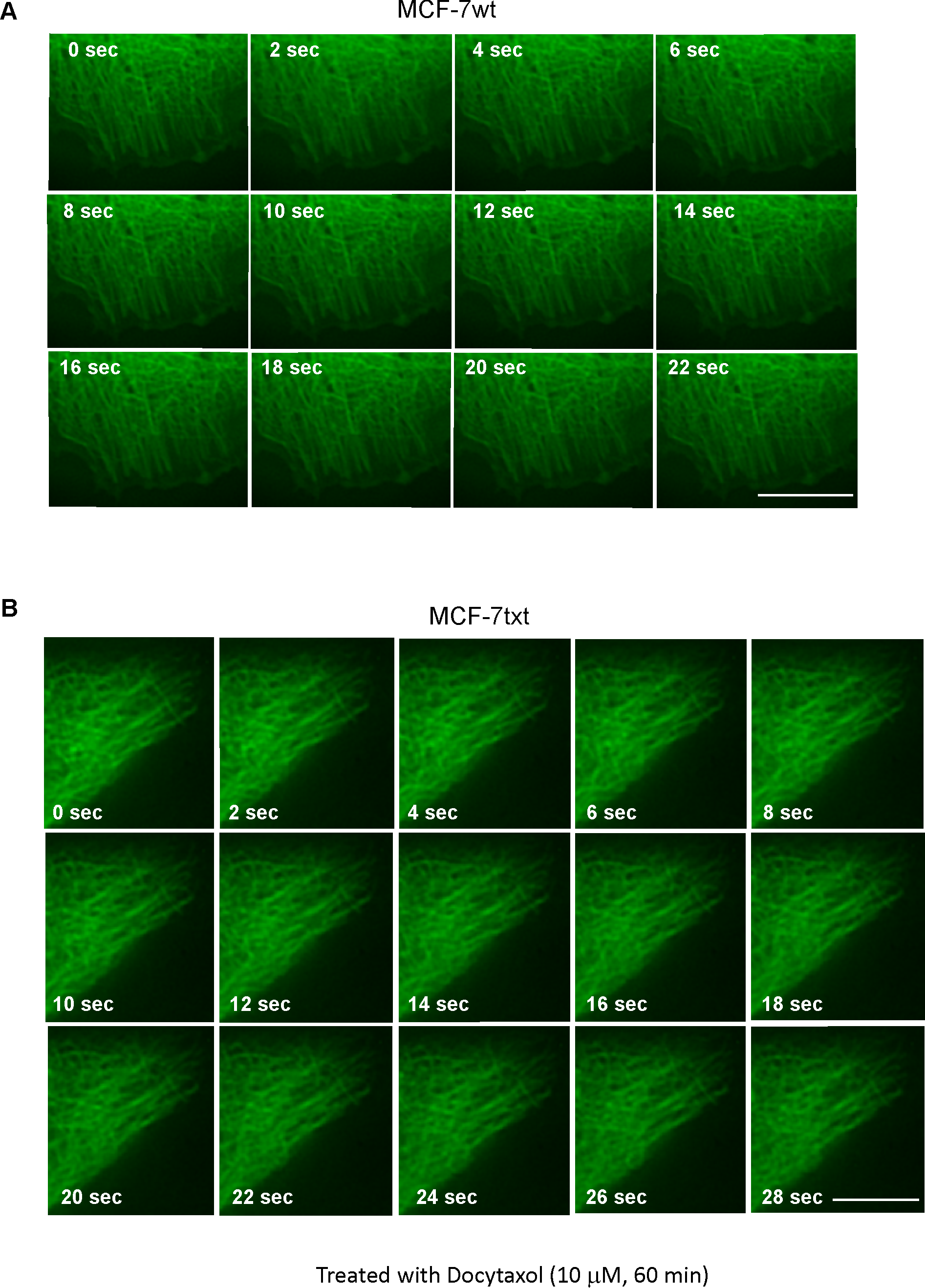

Supplement: Additional file 5: Figure S3 — Selected images from the live imaging (Additional file 3: Video S6&7) of microtubule dynamics of MCF-7wt (A) and MCF-7txt (B) cells following treatment with 10 μM docetaxel for 1 hour. Arrow indicates the extending microtubules. Arrow head indicates the shortening microtubules. Size bar, 10 μm. [file 1471-2407-14-37-S5.tiff]
